# The *In Vivo* Fibrotic Role of FIZZ1 in Pulmonary Fibrosis

**DOI:** 10.1371/journal.pone.0088362

**Published:** 2014-02-06

**Authors:** Tianju Liu, Hongfeng Yu, Matthew Ullenbruch, Hong Jin, Toshihiro Ito, Zhe Wu, Jianhua Liu, Sem H. Phan

**Affiliations:** Department of Pathology, University of Michigan Medical School, Ann Arbor, Michigan, United States of America; University of Kentucky, United States of America

## Abstract

FIZZ (found in inflammatory zone) 1, a member of a cysteine-rich secreted protein family, is highly induced in lung allergic inflammation and bleomycin induced lung fibrosis, and primarily expressed by airway and type II alveolar epithelial cells. This novel mediator is known to stimulate α-smooth muscle actin and collagen expression in lung fibroblasts. The objective of this study was to investigate the in vivo effects of FIZZ1 on the development of lung fibrosis by evaluating bleomycin-induced pulmonary fibrosis in FIZZ1 deficient mice. FIZZ1 knockout mice exhibited no detectable abnormality. When these mice were treated with bleomycin they exhibited significantly impaired pulmonary fibrosis relative to wild type mice, along with impaired proinflammatory cytokine/chemokine expression. Deficient lung fibroblast activation was also noted in the FIZZ1 knockout mice. Moreover, recruitment of bone marrow-derived cells to injured lung was deficient in FIZZ1 knockout mice. Interestingly in vitro FIZZ1 was shown to have chemoattractant activity for bone marrow cells, including bone marrow-derived dendritic cells. Finally, overexpression of FIZZ1 exacerbated fibrosis. These findings suggested that FIZZ1 exhibited profibrogenic properties essential for bleomycin induced pulmonary fibrosis, as reflected by its ability to induce myofibroblast differentiation and recruit bone marrow-derived cells.

## Introduction

Found in inflammatory zone (FIZZ1), initially identified in lung allergic inflammation, belongs to a class of cysteine-rich secreted proteins known as the FIZZ/RELM (resistin-like molecules) family [1], [2], [3]. The FIZZ/RELM family consists four known members, FIZZ1/RELMα, FIZZ2/RELMβ, FIZZ3/resistin, and RELMγ, characterized by their conserved 10-cysteine residue motif, with 2 members, FIZZ2/RELMβ and FIZZ3/resistin, having an additional cysteine residue in the highly variable N-terminus [1], [2], [3], [4]. This family has a unique tissue expression pattern. FIZZ1 is prominently expressed in white adipose tissue with low levels of expression noted in lung, heart and mammary glands. FIZZ1 is first reported to inhibit the nerve growth factor-mediated gene expression in dorsal root ganglion neurons [1]. It has an inhibitory effect on 3T3-L1 preadipocyte differentiation into adipocytes, but does not affect cell proliferation [5]. Together with Ym1, FIZZ1 is specifically produced by macrophages in response to IL-4 both in vivo and in vitro [6], [7], and have been used as indicators of alternative activation. Recently, more evidence showed that FIZZ1 is involved in various lung diseases. FIZZ1 is highly induced in a mouse chronic hypoxia model of pulmonary hypertension, where it is referred to as hypoxia-induced mitogenic factor (HIMF) [8]. FIZZ1 has multiple functions including mitogenic, angiogenic and vascular remodeling roles [8], [9], [10]. FIZZ1 is highly induced in bleomycin (BLM)-induced lung fibrosis [11], [12]. In the lung it is primarily expressed by airway and alveolar epithelial cells (AEC), which is upregulated by the Th2 cytokines IL-4 and IL-13 via STAT6 dependent mechanisms. In vitro FIZZ1 stimulates type I collagen and α-smooth muscle actin (α-SMA) expression in lung fibroblasts indicative of myofibroblast differentiation, a key feature in lung fibrosis. Similar effects are noted during airway remodeling in a model of allergic airway disease [13]. Additionally FIZZ1 has an anti-apoptotic effect on lung fibroblasts in vitro [11], [12], [14], [15]. Induction of FIZZ1 has also been noted in a murine model of colitis, where it is found to recruit inaflammatory cells and is chemotactic for eosinophils [16], consistent with its ability to recruit bone marrow derived cells to the lung [17], [18]. In the gut it is reported to also activate IL-17 pathogenic responses and inflammation [19].

Idiopathic pulmonary fibrosis (IPF) is a devastating condition that leads to progressive lung destruction and scarring. It is currently believed that IPF is an epithelial-fibroblastic disease, in which unknown factors disrupt the homeostasis of alveolar epithelial cells, resulting in diffuse epithelial cell injury with aberrant epithelial repair and activation [20]. Profibrotic mediators, such as TGF-β1, produced by these injured AEC can then recruit and activate adjacent mesenchymal cells to constitute fibrotic foci composed of fibroblast-like cells, including differentiated myofibroblasts [20], [21], [22], which are the key effector cells in fibrogenesis [23], [24]. Given that FIZZ1 is primarily induced in AECs and functions as an inducer of myofibroblast differentiation it is likely to play a significant role in mediating such cross-talk between epithelial cells and fibroblastic cells.

The objective of this study was to investigate the in vivo role of FIZZ1 in pathogenesis of BLM-induced pulmonary fibrosis. Further elucidation of the in vivo importance of FIZZ1 induction should contribute to understanding of key processes involved in progressive fibrosis. FIZZ1 knockout mice were created to evaluate the effects of FIZZ1 deficiency on BLM-induced pulmonary fibrosis. The FIZZ1 deficient mice showed significant impairment of pulmonary fibrosis in response to BLM treatment. Moreover induced FIZZ1 overexpression using an adenoviral construct exacerbated fibrosis thus confirming that FIZZ1 is a profibrogenic factor in pulmonary fibrosis.

## Materials and Methods

### Animals and Induction of Pulmonary Fibrosis

C57BL/6 mice (8∼10 weeks old) and GFP transgenic mice on C57BL/6 background (stock# 003291) were purchased from The Jackson Laboratory (Bar Harbor, ME). To induce pulmonary fibrosis, BLM (Blenoxane, Mead Johnson, NJ) was dissolved in sterile PBS and instilled endotracheally on day 0 at a dose of 2 U/kg body weight into mice as before [25]. Control groups received the same volume of sterile PBS only. Animals (n = 3∼5) were randomly assigned to each of the indicated treatment groups. At indicated time points after BLM treatment, the mice were sacrificed and the lungs were harvested rapidly. All animal studies were reviewed and approved by the Committee on Use and Care of Animals at the University of Michigan. Where indicated, bronchial-alveolar lavage fluid (BALF) was collected by lavaging the lung, and total cell number from the pooled lavages was counted using a hematocytometer. BAL cells were then cytospun and stained with Quick Diff (IMEB Inc., San Marcos, CA) for differential cell count. At least 500 cells were counted in 10 high-power (400×) fields.

### Generation of FIZZ1 Traditional Knockout (KO) Mice

**Figure 1:**
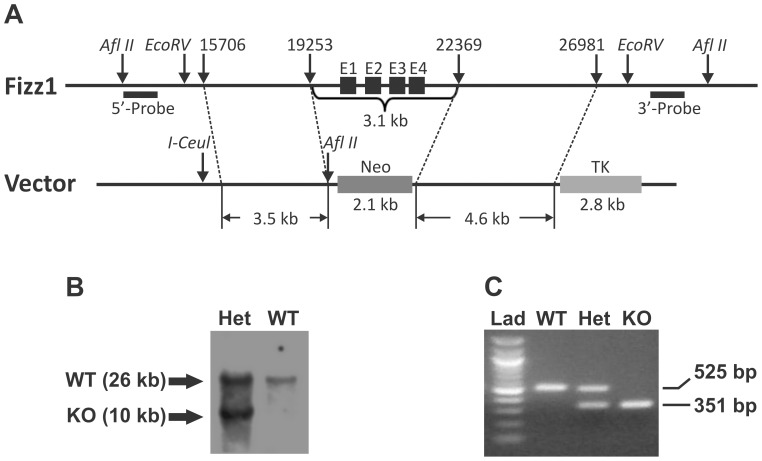
Generation of FIZZ1 KO mice. (A) Gene targeting strategy and restriction map of FIZZ1 gene. The diagram showed the wild type FIZZ1 gene allele and the gene targeted allele. The black boxes E1–E4 represent the four exons of FIZZ1 gene. All four exons of the FIZZ1 gene were replaced by a Neo gene cassette. (B) Southern blotting analysis of an ES cell clone with homologous recombination. A 3′ probe (shown in A) was used to detect 26 kb WT and 10 kb KO alleles. (C) PCR genotype using mouse tail DNA from wild type (“WT”), FIZZ1 heterozygote (“Het”) and homozygous knockout (“KO”) mice. The PCR fragment for WT was 525 bp, and KO was 351 bp. “Lad” referred to 100 bp ladder.

FIZZ1 targeting vector pVBTK-FIZZ1 was custom ordered from Vega Biolab (Eagleville, PA) (Figure 1A). The FIZZ1 5′ homology arm (3.5 kb) that is upstream from exon 1 and the 3′ homology arm (4.6 kb) that is downstream of exon 4 (129 s6/SvEv mouse DNA) were inserted into targeting vector pVBTK containing a neomycin resistance gene. This targeting vector was confirmed by restriction enzyme digestion prior to linearization (by I-Ceu) and electroporation into W4 ES cells. A region of 3.1 kb including all four FIZZ1 exons was replaced by a neomycin gene cassette. ES cell clones with successful homologous recombination were screened by Southern blotting with Afl II digestion. Wild type (WT) and KO alleles generated 26 kb and 10 kb fragment, respectively (Figure 1B). The 449 bp Southern blotting probe that located downstream of the 3′ homology arm was generated by PCR (forward primer 5′-TCTGGTCACACTTGCATTCC-3′; reverse primer 5′-AAAAAGGTGTGCGTTCCATT-3′). Three independent homologous-recombinant ES clones were injected into C57 BL/6 blastocysts and chimera mice were generated. Heterozygous were produced by breeding these chimera mice with C57 BL/6 WT mice. Genotyping was identified by PCR (Figure 1C). Primers for a 525 bp FIZZ1 fragment in WT allele were: forward primer 5′-GAATGGGTGGGTAGGGAAGT-3′, and reverse primer 5′-CAGGAAGAGCTGGCATAAGG-3′. Primers for a 351 bp neomycin fragment in KO allele were: forward primer 5′-GTGCTCGACGTTGTCACTGAAGCGG-3′, and reverse primer 5′-GATATTCGGCAAGCAGGCATCG-3′. Heterozygous offspring was backcrossed to C57BL/6 WT mice for at least 7 generations. Homozygous KO mice were generated by breeding the heterozygous KO mice. ES homologous recombination, blastocyst microinjection, and generation of chimera mice were performed by the Transgenic Core at the University of Michigan. Pulmonary fibrosis was induced by endotracheal BLM injection as described above. Where indicated, blood was collected from the heart, and serum samples were then prepared using serum separator tubes (BD Biosciences, San Jose, CA).

### Generation of GFP-bone Marrow (GFP-BM) Chimera Mice

GFP-bone marrow (BM) chimeras were generated by transplantation of BM cells from GFP transgenic mice to lethally irradiated WT recipient mice as previously described [26]. Six weeks after transplantation, pulmonary fibrosis was induced by endotracheal BLM injection as described above, and 21 days later the lungs were removed for analysis of GFP^+^ BM cell recruitment.

### Construction of Adenoviral FIZZ1

**Figure 2:**
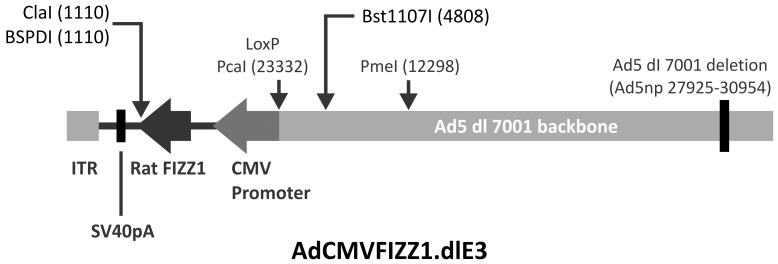
Construction of AdFIZZ1. AdCMVFIZZ1.dlE3 was generated by inserting a 500-LoxP recombination between shuttle vector and the cAd5-deltaE3.LoxP cosmid. Rat FIZZ1 cDNA was under CMV promoter. “ITR” referred to the Ad5ITR and Packaging signal.

The rat FIZZ1 cDNA fragment (∼500 bp) was inserted into Hind III and EcoRI sites of the Ad5 shuttle vector pACCMV2. A full-length E1, E3 deleted recombinant adenovirus was then generated (Figure 2) in conjunction with the Vector Core of the University of Michigan using in situ loxP recombination between the shuttle vector (linearized by PmeI) and cAd5-deltaE3.LoxP cosmid containing the Ad5 backbone (linearized by ClaI) in the presence of Cre recombinase [27]. The resulting recombinant adenoviral DNA was then transfected into HER911 cells. Recombinant clones were identified as plaques in soft agar culture, and then verified by real time RT-PCR. Large scale high titer adenoviral purification, particle determination (particles/ml), and titer determination (plaque formatting units (pfu)/ml) were performed in the Vector Core. 10^8^ pfu/rat was endotracheally injected into rats alone or combined with BLM. Empty adenovirus without FIZZ1 (AdCont) were used as control.

### Isolation of Lung AECs and Fibroblasts

Rat type II alveolar epithelial cells (AECs) were isolated by elastase digestion and IgG panning as previously described [28]. The cells were suspended in DMEM supplemented with 10% fetal bovine serum (FBS, Sigma-Aldrich, St. Louis, MO), and then plated onto 6-well tissue culture dishes pre-coated with fibronectin (R&D Systems, Minneapolis, MN). Fibroblasts were isolated from lung tissues and maintained in culture as described previously [25]. Fibroblasts between passages 3 to 5 after primary culture were used.

### qPCR and Western Blotting Analysis

Total RNA was isolated from lung fibroblasts, AECs and lung tissues after indicated treatments. The following primers and probes were used: rat α-SMA: forward primer (5′-GTGGAGGTTGTTGCCAATATGAT-3′), reverse primer (5′-TGATGATGCCGTGTTCTATCG-3′), and probe (6FMA) (5′-CCTGACCCTGAAGTAT-3′); mouse α-SMA forward primer (5′-CTGGAGAAGAGCTACGAACTGC-3′), reverse primer (5′ CTGATCCACATCTGCTGGA AGG-3′), and probe (FAM) (5′ CTGACGGGCAGGTGA-3′). Primers and probes for FIZZ1, type I procollagen, MCP-1, TERT, IL-4 and GAPDH were purchased from Applied Biosystems. One-step RT-PCR was performed by using 100 ng of total RNA as template. The mRNA levels were normalized to GAPDH signal.

Total protein was isolated from cultured mouse lung fibroblasts (MLF) and snap-frozen mouse lung tissues using RIPA lysis buffer (Pierce, Rockford, IL). The following primary antibodies were used: anti-rat FIZZ1 (Alexis Biochemicals, San Diego, CA), anti-α-SMA (Sigma), anti-rat or mouse type I collagen (Biodesign International, Saco, ME), and HRP-conjugated anti GAPDH (Abcam, Cambridge, MA).

### Hydroxyproline Assay

Lung collagen deposition was estimated by measuring the hydroxyproline (HYP) content of whole lung homogenates as previously described [29], [30]. The results were expressed as µg HYP per lung.

### BM Cell Isolation and Migration Assay

Mouse BM cells were isolated from femurs and tibias by aspiration and flushing. Where indicated, BM-derived dendritic cell (BMDC) were induced with 10 ng/ml GM-CSF (R&D Systems), and purified with MACS system using CD11c microbeads (Miltenyi Biotec, Auburn, CA). Both CD11c+ and CD11c− cells were collected where indicated. For cell migration assay, 1×10^6^ BM or BMDC cells were pre-stained with a fluorescent dye Calcein AM (BD Biosciences), and then placed into inserts with 5 µm of pore size in 24-wel transwell Boyden chamber (Fisher Scientific, Waltham, MA). FIZZ1 was added into the lower chamber at the indicated concentration. After 2 hours of incubation, fluorescence was read at Ex494/Em517 using Spectramax Gemini XS Microplate Spectrofluorometer (Molecular Devices, Menlo Park, CA).

### Flow Cytometry Analysis

The lung cells were stained with CD11c-PE conjugates (BD Biosciences), and then CD11c and GFP positive cells were analyzed by FACS Caliber flow cytometer (BD Biosciences).

### Statistical Analysis

All data were expressed as mean±SE unless otherwise indicated. Differences between means of various treatment and control groups were assessed for statistical significance by ANOVA followed by *post hoc* analysis using Scheffé’s test for comparison between any two groups. A P value <0.05 was considered to indicate statistical significance.

## Results

### FIZZ1 Deficiency Impaired BLM-induced Pulmonary Fibrosis

FIZZ1 KO mouse was generated on C57BL/6 background as described in the Methods. These FIZZ1 KO mice were fertile and did not show gross anatomic abnormalities compared with their WT littermates. There was no significant difference between KO and WT littermates in their body weights, major organ weights, blood cell counts, as well as some serum chemistries tested including glucose, triglycerides, insulin, lipase, albumin, etc (data not shown).

**Figure 3:**
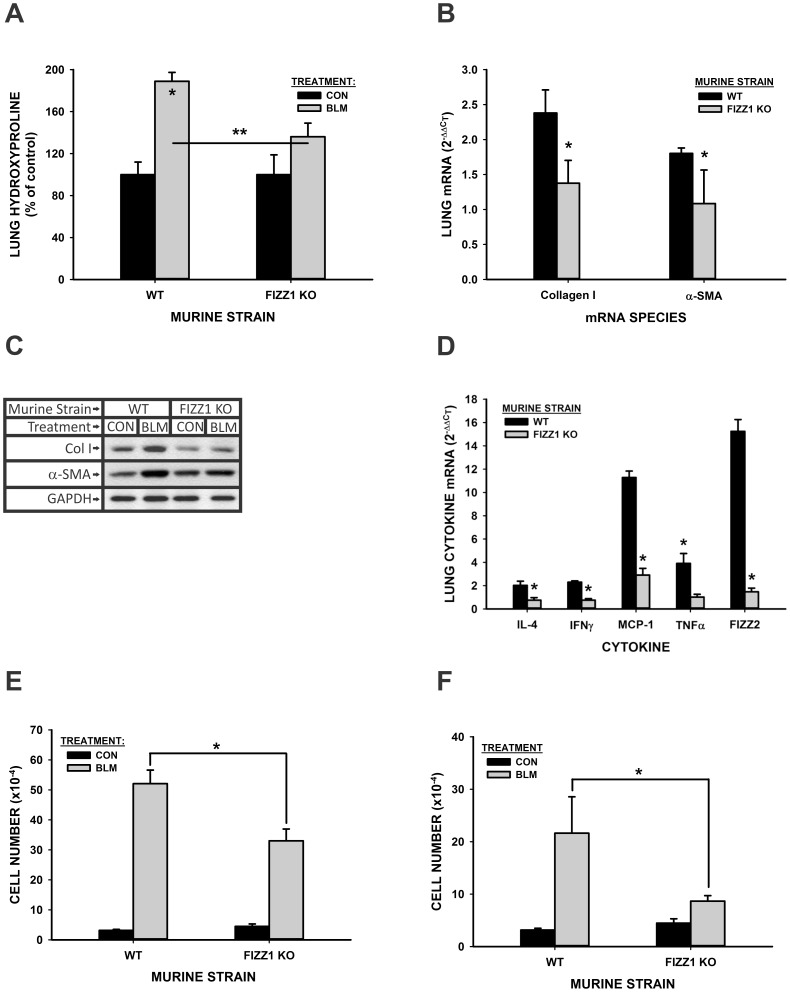
Effects of FIZZ1 deficiency on BLM-induced pulmonary fibrosis. WT control or FIZZ1 KO mice were treated with PBS (CON) or BLM as indicated. The lungs were harvested at day 21, and analyzed for lung HYP content (A). The values were expressed as percentages of their respective PBS control. Data were shown as mean ± SE with 5 mice in each group. *P<0.05 or **P<0.001 compared to control. Type I collagen, α-SMA and mRNA and protein in the lungs were also analyzed by qPCR (B) and Western blotting (C), respectively. A typical blot from 5 mice in each group was shown. The lung RNA from day 7 after PBS (CON) or BLM treatment was also analyzed for cytokines mRNA expression by qPCR (D). Results were expressed as 2^−ΔΔCT^. The values were expressed as percentages of their respective PBS control. The numbers of total BAL cell (E) and BAL macrophage/monocyte (F) were counted at day 7 of BLM or PBS treated WT or FIZZ1 KO mice. Data were shown as mean ± SE (n = 7 mice for total BAL cell, 4 mice for macrophage/monocyte).

To investigate further the implicated profibrogenic role of FIZZ1 in vivo, the effects of FIZZ1 deficiency on BLM-induced pulmonary fibrosis were evaluated. As expected, WT mice exhibited significant BLM-induced pulmonary fibrosis with more than 80% elevation in whole lung collagen content as determined by hydroxyproline (HYP) assay at day 21 after BLM injection (Figure 3A). This increase was significantly reduced to <30% in FIZZ1 KO mice, which was not statistically significant relative to the PBS-treated KO mice. Furthermore lung type I collagen and α-SMA mRNA (Figure 3B) and protein (Figure 3C) levels showed similar differences between WT and KO mice. Thus significant BLM-induced increases in type I collagen and α-SMA expression in WT mice were significantly reduced in KO mice after BLM injection, consistent with the reduced fibrosis noted on the basis of lung HYP content. Moreover the reduced α-SMA suggested significantly reduced myofibroblast differentiation in the FIZZ1 KO mice. The effects on lung inflammatory and fibrogenic cytokine expression revealed significant reduction as well in the FIZZ1 KO mice when compared to the WT responses to BLM treatment. Thus the expected BLM induction of all cytokines analyzed (IL-4, IFN-γ, MCP-1, TNF-α, FIZZ2) observed in WT mice was markedly diminished in FIZZ1 KO mice (Figure 3D). Of special note were dramatic reductions in MCP-1 and FIZZ2 mRNA levels (>70% inhibition). These significant reductions in fibrosis and cytokine expression were accompanied by significant reduction in BLM-induced increase in accumulation of BAL cells. The total BAL cell (Figure 3E) and macrophage (Figure 3F) numbers counted at day 7 after BLM treatment were significantly elevated relative to those in PBS treated controls in WT as expected, but were significantly reduced in FIZZ1 KO mice. These results indicated that FIZZ1 deficiency significantly reduced pulmonary fibrosis, possibly by reducing lung myofibroblast differentiation, inflammatory cell recruitment and inflammatory/fibrogenic cytokine expression.

### FIZZ1 Recruitment of BM-derived Cells and Macrophages

**Figure 4:**
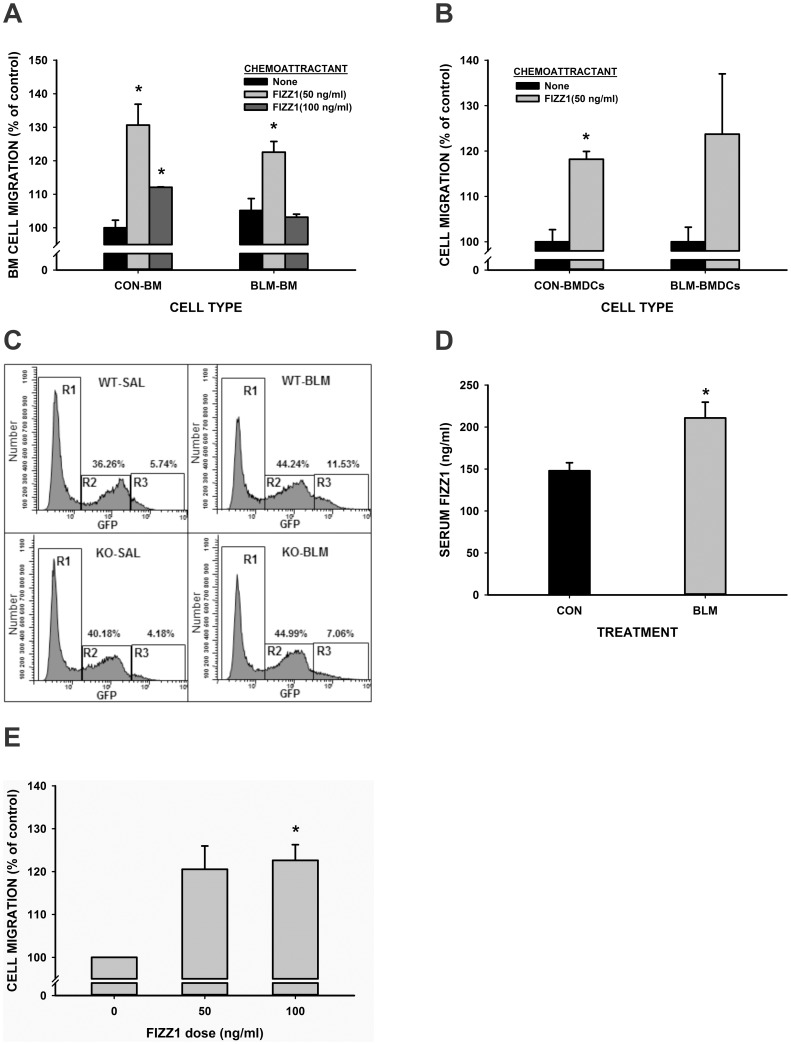
FIZZ1 effects on BM cell recruitment. (A) Fresh isolated whole BM cells from day 7 BLM (“BLM-BM”) or PBS (“CON-BM”) treated mice were preloaded with fluorescent dye, and then plated into 5 µm-inserts in 24-well transwell plates. After 2 hours of incubation with the indicated doses of FIZZ1 in the lower chambers, the cells that have migrated to the lower chambers were quantitated by measuring the fluorescence with an excitation and emission wavelengths of 494 and 517 nm, respectively. The results were normalized to the controls and expressed as percentages of controls, and shown as mean ± SE (n = 3). In (B) purified BM-derived DC from day 7 BLM (“BLM-BMDC”) or PBS (“CON-BMDC”) treated mice were similarly analyzed as in (A) for migration to media only (“None”) or to 50 ng/ml FIZZ1 in the lower chambers. The results were expressed as in (A). (C) BM from GFP transgenic mice were transplanted into WT or FIZZ1 KO mice to create GFP BM chimera mice of the respective recipient strain. After stable engraftment the mice were treated with BLM and 7 days later were analyzed for GFP expression in the lung cell population by flow cytometry. Three populations were discerned corresponding to the cells with undetectable (R1), low (R2) or high (R3) GFP fluorescence. One representative data was shown from two individual experiments, and the combined lung cells from two mice were used for flow cytometry in each group. Circulating FIZZ1 protein was measured in the sera (1∶50 dilution) by ELISA assay 21 days after BLM or PBS treatment (D). Results were shown as mean ± SE with 5–6 mice in each group. (E) Effect of FIZZ1 on migration of primary cultured mouse lung fibroblasts was also analyzed as described in legend for (A). *P<0.05 compared to control.

BM derived cell recruitment to the lung is essential for fibrotic responses in that tissue [26], [31], [32], [33], [34], [35]. Some of these cells express type I collagen, c-kit and TERT. FIZZ1 was recently reported to have a chemotactic effect on eosinophils and macrophages [16] and its overexpression in alveolar epithelial cells recruits CD11c^+^ cells to the lung [18]. To evaluate a possible role of FIZZ1 in recruitment of BM-derived cells in BLM-induced pulmonary fibrosis, whole mouse BM cells isolated at 7 days after BLM treatment were analyzed for their migratory response to FIZZ1 in a Boyden chamber assay. The results showed significant migratory activity to FIZZ1 by BM cells isolated from both PBS and BLM-treated animals (Figure 4A). Interestingly, migratory activity was greater in the cells from PBS treated control mice, suggesting either desensitization from prior in vivo stimulation by the induced FIZZ1 in BLM-treated mice, or the depletion of responsive cells due to prior recruitment to the lung in vivo as a result of BLM treatment, as previously suggested [35]. This migratory response to FIZZ1 remained even after the BM cells were treated with GM-CSF to induce differentiation to CD11c^+^ dendritic cells (BMDCs) (Figure 4B). To evaluate the in vivo relevance of these findings, the effect of FIZZ1 deficiency on BM recruitment to the lung was assessed in the BLM model using GFP BM chimera mice to allow tracking of BM cell movement using their GFP marker (20). Analysis of lung cells at day 7 after PBS or BLM injection revealed the presence of two distinct subpopulations of GFP-positive cells, one with low level GFP expression (R2 in Figure 4C) and another with high GFP expression (R3 in Figure 4C). BLM treatment caused an increase only in the high GFP subpopulation while the low GFP subpopulation remained unchanged. However this BLM-induced increase in the high GFP subpopulation was not observed in FIZZ1 KO mice, which had also been similarly transplanted with BM from GFP transgenic mice with intact FIZZ1 gene. Thus FIZZ1 expression by the recipient mice was essential for recruitment of BM cells. At day 14 after BLM injection similar reduction in the number of high GFP cells in FIZZ1 KO lungs was seen in comparison with BLM injected WT lungs (data not shown). Based on the forward light scatter, these high GFP expressing cells appeared to be larger than the low GFP expressing cells. Thus BLM-induced lung FIZZ1 expression played a role in the recruitment of BM cells. This was consistent with the results from analysis of circulating FIZZ1 level in the FIZZ1 KO mouse serum, which showed significant elevation (42.5% above that in PBS controls in circulating FIZZ1 levels in BLM treated WT mice at day 21 (Figure 4D). This elevation in circulating FIZZ1 could mediate the recruitment of the BM cells into the lung. In contrast FIZZ1 was undetectable in serum samples from both PBS and BLM-treated FIZZ1 KO mice, thus confirming FIZZ1 deficiency in the KO mice with consequent impairment of BM cell recruitment to the lung (Figure 4C). Given that FIZZ1 was chemotactic to BMDCs (Figure 4B), the high GFP positive cells were examined for their expression of CD11c by flow cytometry. The results revealed that >95.53% of this population were positive for CD11c (data not shown). Finally, the possible migratory effect of FIZZ1 on lung fibroblasts was evaluated as described for BM cells. The data showed that FIZZ1 also stimulated fibroblast migration (Figure 4E), suggesting a potential role in formation of fibroblastic foci.

**Figure 5:**
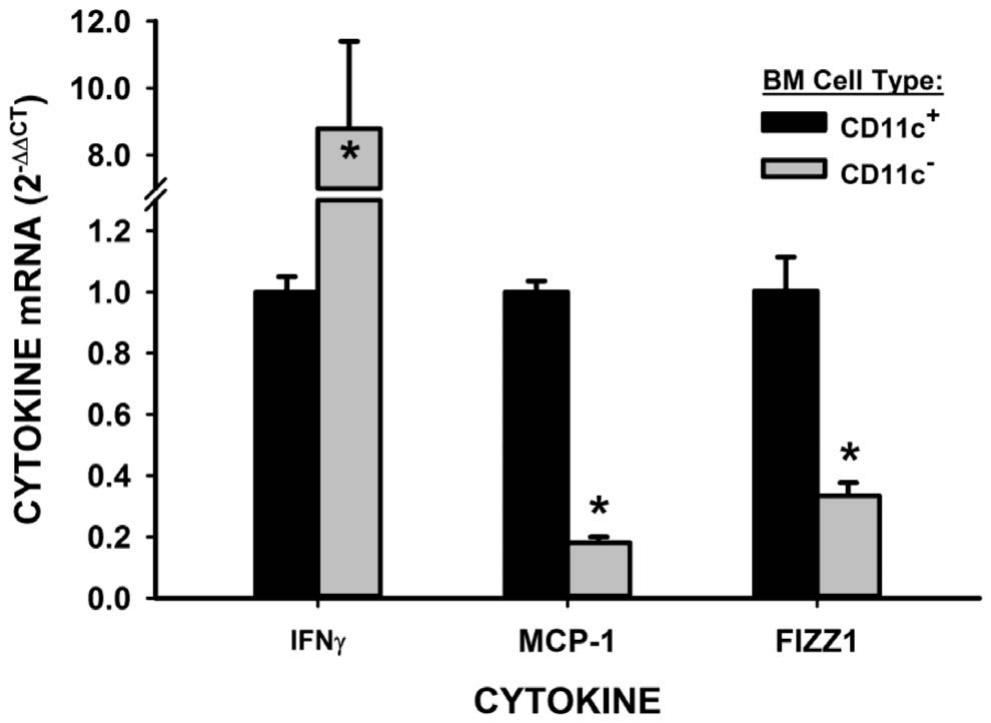
Cytokine expression in BMDC. GM-CSF-induced BMDC were separated by MACS with anti-CD11c microbeads. IFNγ, FIZZ1 and MCP-1 were detected in two distinct cell populations. *P<0.05 vs. respective controls.

To evaluate if BMDCs could be a source of the induced lung cytokine expression associated with this BLM model, the expression of MCP-1, FIZZ1 and IFNγ were evaluated in CD11c+ BMDCs. The results showed that the profibrogenic cytokines MCP-1 and FIZZ1 were expressed mainly in CD11c^+^ BMDCs with much lower levels of expression in the CD11c negative BM cells (Figure 5). In contrast expression of the antifibrogenic cytokine IFNγ was predominantly in CD11c negative BM cells, being >8-fold higher than in CD11c^+^ BMDCs (Figure 5).

**Figure 6:**
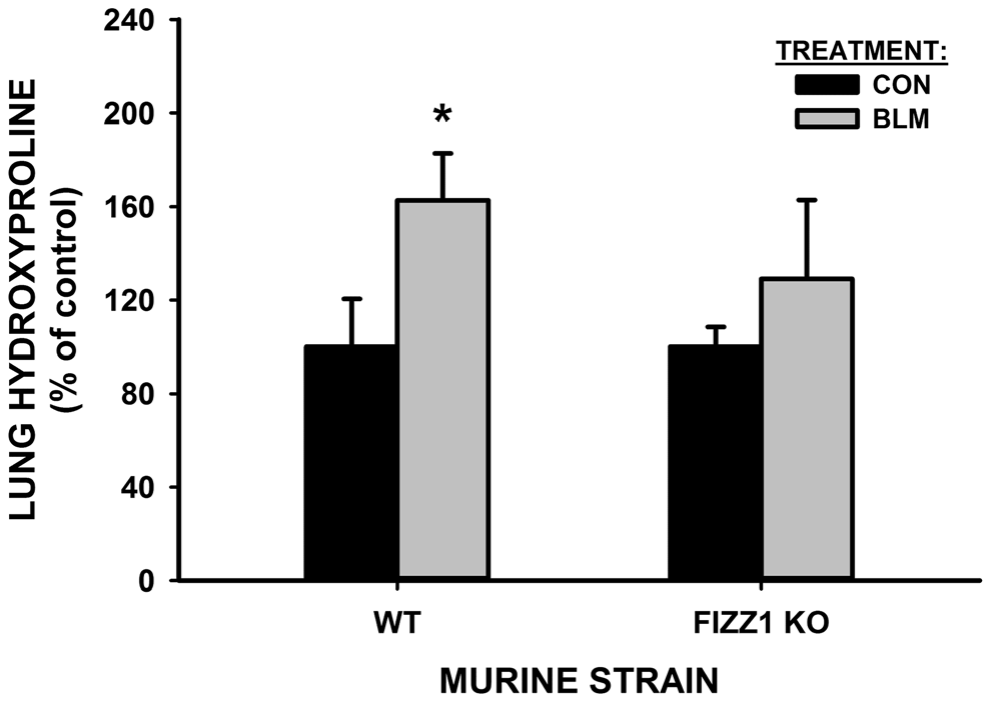
The lung HYP content was measured in GFP-FIZZ1 chimera lung samples after BLM or PBS treatment. Data were shown as percentages of their respective PBS controls. The mean values were shown from 2 mice each group. *P<0.05 vs. respective control values.

Since these studies of BM cell recruitment to the lung were conducted using donor GFP transgenic mice with intact FIZZ1 gene (i.e. wild type with respect to FIZZ1), they afforded the opportunity to evaluate the effect of WT BM reconstitution on BLM-induced fibrosis in FIZZ1 KO mice. The results showed that transplantation of WT BM from GFP mice failed to significantly alter the deficient pulmonary fibrosis in the FIZZ1 KO recipient mice (Figure 6). These results together demonstrated that FIZZ1 deficiency impaired BM cell recruitment and pulmonary fibrosis, which could not be corrected by transplantation of wild type BM since lung-derived FIZZ1 remained deficient.

### FIZZ1 Overexpression Induced Myofibroblast Differentiation and Pulmonary Fibrosis

**Figure 7:**
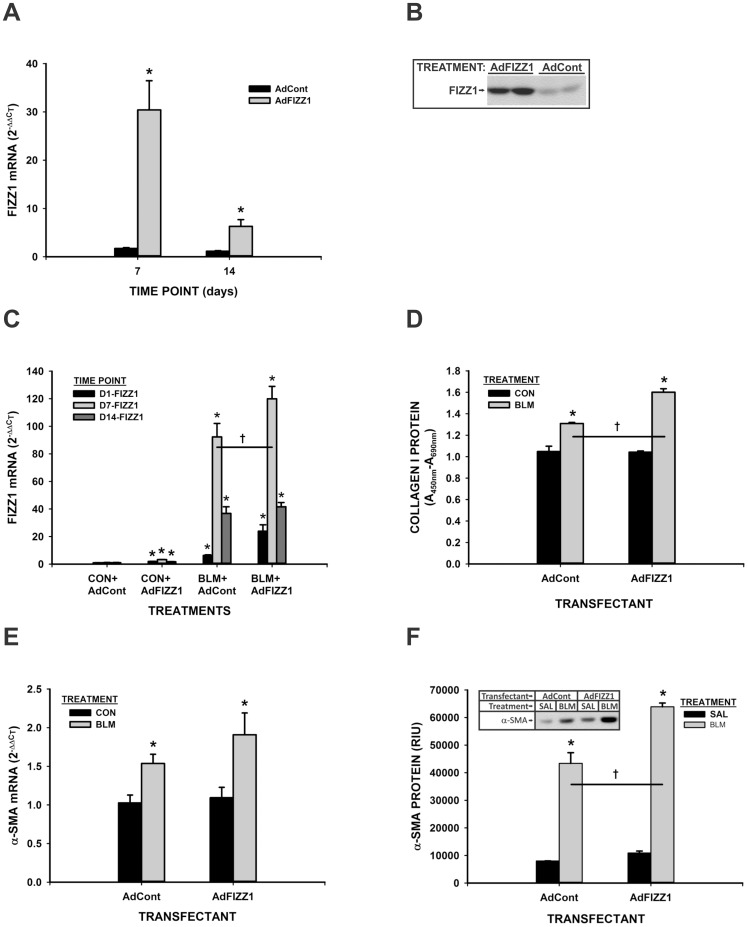
AdFIZZ1 effects on lung fibrosis. The lung FIZZ1 mRNA (A) at the indicated time points and protein at day 21 (B) after AdFIZZ1 endotracheal administration alone, or with BLM injection (C) were analyzed by qPCR. The results were shown as mean ± SE of triplicate samples or animals. Type I collagen production in the lungs was analyzed at day 7 after indicated treatments with 200 ng lung tissue lysates by ELISA (D). Data were shown as mean ± SE with 5 samples in each group. Lung α-SMA mRNA at day 7 (E) and protein (F) after AdFIZZ1 injection with BLM or PBS were analyzed by qPCR or Western blotting, respectively. A representative blot was shown in (F).*P<0.05 compared to their respective controls. *P<0.05 or †P<0.001 compared to control.

To further confirm the importance of FIZZ1 in myofibroblast differentiation and pulmonary fibrosis, a FIZZ1 adenovirus (AdFIZZ1) was produced to evaluate its effects in vivo. Endotracheal injection of AdFIZZ1 (10^9^ pfu) caused significant up regulation in lung tissue FIZZ1 mRNA, which persisted up to as long as 14 days after injection (Figure 7A). Consistent with mRNA induction, FIZZ1 protein was also induced by AdFIZZ1 injection at day 21 (Figure 7B). This enhanced FIZZ1 expression was not seen with injection of control adenovirus (AdCont). When animals treated with BLM plus AdFIZZ1 (10^8^ pfu), significant enhancement in FIZZ1 expression was noted at days 1 and 7, which became insignificant on day 14 after BLM treatment (Figure 7C). This enhancement of lung FIZZ1 expression was accompanied by significant enhancement in type I collagen expression (Figure 7D) as well as α-SMA mRNA and protein levels (Figures 7E and 7F, respectively). These findings complemented the results using FIZZ1deficient mice by demonstrating that FIZZ1 overexpression could enhance fibrosis. FIZZ1 had no significant effects on type II alveolar epithelial cells (AEC II) expression of α-SMA, collagen I and the epithelial cell marker E-cadherin, or apoptosis (data not shown). Thus FIZZ1 did not induce epithelial-mesenchymal-transition (EMT) or alter epithelial cell survival in mediating its effects on fibrosis.

## Discussion

Multiple lung disorders, including the various interstitial lung diseases, can result in significant chronic progressive fibrotic lung disease, and in certain cases such as IPF, it is a progressive and fatal disorder. Fibrotic lesions are characterized by the presence of fully differentiated myofibroblasts, which are a key cellular source of extracellular matrix [20], [21], [24]. In the case of IPF, factors produced by repeatedly injured alveolar epithelial cells are thought to be responsible for recruiting adjacent fibroblasts to form distinct fibrotic foci containing myofibroblasts. Therefore understanding the potential role of such factors that may mediate interaction between epithelial cells and fibroblasts/myofibroblasts in the formation and expansion or progression of fibrotic foci may be insightful in understanding the overall pathogenesis of these fibrotic disorders. Given that FIZZ1 is a cytokine secreted mainly by alveolar epithelial cells, it may function as an effector cytokine mediating the communication between injured epithelial cells and fibroblasts. In addition to TGFβ1, FIZZ1 is recently identified as another inducer of myofibroblast differentiation in pulmonary fibrosis. In the present study, we demonstrated that FIZZ1 was able to induce pulmonary fibrosis in vivo through induction of myofibroblast differentiation, which is consistent with its in vitro function on myofibroblast differentiation [11]. BLM-induced lung fibrosis was impaired in FIZZ1 deficient animals, while its overexpression exacerbated fibrosis. FIZZ1 was also found to promote migration of BM cells in vitro, and its deficiency in vivo resulted in diminished recruitment of BM derived cells into BLM-injured lungs. Since BM cells are essential for pulmonary fibrosis [26], [33], FIZZ1-mediated BM cell recruitment to the lung might represent an additional mechanism by which it could promote fibrosis.

FIZZ1 is reported to play significant roles in pulmonary inflammation, airway and vascular remodeling in animal models of allergic inflammation and pulmonary hypertension via upregulating proinflammatory mediators, such as MCP-1, and recruiting inflammatory cells such as macrophages/monocytes in the lung [9], [10], [13]. FIZZ2 (or RELMβ), another FIZZ/RELM family member, is also shown to promote airway and lung remodeling in the mouse lung by increasing perivascular and lung collagen deposition [29], [36]. These findings suggest that FIZZ1 is a proinflammatory and profibrogenic cytokine in many different inflammatory and remodeling processes. Moreover FIZZ1 has an anti-apoptotic effect on mouse lung fibroblasts mediated in part through ERK signaling [15], [37], thus promoting genesis of the myofibroblast by preservation or enhanced survival of its progenitors. Additionally, FIZZ1 has the capacity to promote lung cell proliferation, including pulmonary microvascular endothelial cells and smooth muscle cells via an AKT-dependent pathway [8], [9]. Both these functions of FIZZ1 should result in enlarging the pool of cells with the capacity for activation by FIZZ1 itself plus other factors, such as TGFβ, and subsequent differentiation to myofibroblasts.

An additional role for FIZZ1 is suggested by its reported ability to promote migration of BM-derived cells in conjunction with evidence for the importance of such recruited cells in pulmonary fibroproliferative and vascular disorders [17]. Thus intraperitoneal injection of FIZZ1 induces a local inflammatory response and significantly increases the accumulation of eosinophils and lymphocytes, but not neutrophils [16]. Conditional (CCSP-driven) overexpression of FIZZ1 in lung epithelial cells also resulted in recruitment of CD11c^+^ dendritic cells [18]. Consistent with the latter finding, in the present study FIZZ1 was shown to promote migration of CD11c^+^ BMDCs in vitro, while the BLM-induced recruitment of CD11c^+^ cells to the lung was abrogated in FIZZ1 deficient mice, similar to that seen in FIZZ2 deficient mice [29]. Indeed, FIZZ1 can bind to lung CD45+CD11c+ dendritic cells in a fungal allergen *(Alternaria)* induced asthma model, in addition to collagen-1 producing CD45- fibroblasts [38]. FIZZ1 can stimulate the production of stromal cell-derived factor 1 (SDF-1) in lung resident cells [9], suggesting that it could also indirectly attract BM-derived cells by increasing production of chemoattractants (such as SDF-1) in the fibrotic lung. In the present study, BLM-induced increase in numbers of macrophages/monocytes in bronchial-alveolar lavage fluid (BALF) was significantly reduced in FIZZ1 deficient mice, which paralleled the marked reduction of BLM-induced lung MCP-1 expression, one of the most potent chemokines that attract and activate monocytes in many inflammatory disease processes [39]. These findings together suggested that in addition to a direct mechanism, FIZZ1 contribution to the BM-derived cell recruitment, may also be mediated by indirect effects on chemoattractants production, such as MCP-1 and/or SDF-1 in lung resident cells.

In this study, most of the recruited BM-derived cells appeared to be CD11c+ cells, which were the primary source of MCP-1 in BLM-induced pulmonary fibrosis. Moreover FIZZ1 deficiency significantly reduced this recruitment, which was associated with reduction in lung cytokine (including MCP-1) expression and pulmonary fibrosis. These findings suggested that the reduction in BM cell recruitment might be a factor in the reduced fibrogenic cytokine expression with consequent reduction in fibrosis. Such a possibility is supported by previous studies showing the importance of CD11c^+^ DC in fibrosis in diverse tissues. Thus in a mouse model of liver fibrosis, hepatic CD11c^+^ DCs expand 5-fold, inducing TNF-α, MCP-1 and IL-6 expression in co-cultured NK cells, with effects on inflammation, cell proliferation as well as fibrotic response. DC depletion completely abrogates the elevated levels of many inflammatory mediators that are produced in the fibrotic liver [40]. Th2 cytokines IL-4 and IL-13 feedback on the function of the airway DCs and epithelial cells to directly or indirectly (via DC-activating cytokines like TSLP) stimulate DCs in a STAT6-dependent way [41], [42]. Interestingly IL-4 induces alternative activation of BMDC and peritoneal DCs in vitro and in vivo with significantly elevated level of FIZZ1 expression. These IL-4 induced FIZZ1-expressing DCs in turn prime the Th2 response. Moreover this study demonstrates that DC-derived FIZZ1 is required for optimal Th2 cytokine secretion [43]. Additionally, impaired DC function is associated with significant reduction in bleomycin induced pulmonary fibrosis [44], while elevated numbers of DCs in IPF lung have been reported [45]. The totality of these findings provides ample evidence to suggest that expanded numbers of CD11c^+^ DCs in fibrotic tissue could play a role in fibrogenesis.

The present study together with our previous studies [11] suggest that FIZZ1 is a potential profibrogenic cytokine, and was positively associated with the Th2 response. However two recent studies suggest that FIZZ1 is a repressor for the Th2 response [46], [47]. In a mouse lung granuloma model induced by *Schistosoma mansoni* (*Sm*) eggs, FIZZ1 KO mice exhibit elevated expression of pathogen-specific CD4+ T cell-derived Th2 cytokines, including IL-4, IL-5 and IL-13, which is accompanied by increased size of egg-induced granuloma and fibrosis compared with their WT counterparts [46]. Similarly, pulmonary granuloma formation after infection of *Sm* eggs is exacerbated in the absence of *Retlna* gene with enhanced number of granuloma-associated eosinophils [47]. However, another study shows that FIZZ1 expression level is enhanced in exacerbated *Sm* egg-induced granuloma formation in TLR-9 KO mouse [48]. Moreover intranasal administration of recombinant FIZZ1 during granuloma formation significantly enhances collagen deposition in both WT and TLR-9 KO mice, even though there is no clear differences in granuloma size and Th1/Th2 cytokine profile in the granulomatous lungs [48], indicationg the lack of a suppressor effect on either Th1 or Th2 responses. Furthermore, in a dextran sodium sulfate (DSS)-induced colitis model, FIZZ1/RELM-α administration exacerbates colitis and recruits eosinophils to the peritoneum [16]. More recent evidence suggests FIZZ1 leads to airway eosinophilia, increases thickness of the airway epithelium, and peribronchial fibrosis [38]. The basis for these opposite findings on the potential role of FIZZ1 in regulation of Th2 immune responses is unclear, but may be related to differences in experimental approaches and models studied. In this regard, it is noteworthy that the FIZZ1 KO mouse produced in the present study was different from the one used in the two cited studies of pulmonary granuloma models showing a Th2 inhibitor role for FIZZ1 [46], [47],wherein the gene-targeted allele contains a reporter gene, *LacZ*, which was lacking in the FIZZ1 target construct in this study. Thus the expression of β-galactosidase due to the expressed LacZ gene in the KO mice in that study might influence the findings in the studies suggesting a Th2 inhibitor role for FIZZ1 [46]. Further study may be needed to clarify these controversial observations.

The cognate receptor for FIZZ1 on target cells, such as the fibroblast, remains to be identified. Bruton’s tyrosine kinase (BTK) is suggested to play a role in mediating FIZZ1 chemotactic activity for myeloid cells [49] as the functional binding partner of FIZZ1. FIZZ1-induced myofibroblast differentiation requires intact Notch signaling, which appears to be mediated by the induction of the Notch ligand, Jagged1 [14]. Thus diverse pathways appear to be activated upon exposure of a variety of cell types to FIZZ1, which suggest that indirect mechanisms via different ligands may be involved in some of these responses. The human ortholog of rodent FIZZ1 has yet to be identified. Recent reports suggest the possibility that human FIZZ2 may present the human counterpart of rodent FIZZ1, especially at the functional level [50]. Human FIZZ2 is highly induced in the lungs of patients with IPF, and could be up-regulated in human small airway epithelial cell line by Th2 cytokines [29], suggesting a potential role for FIZZ2 in pathogenesis of IPF.

In summary, the present study suggested that FIZZ1 is a multipotential profibrogenic cytokine through induction of myofibroblast differentiation, recruitment of BM-derived cells and induction of other proinflammatory cytokines and chemokines in the cytokine networks.
